# Supplementary figures and images for: Spatial and temporal patterns of human Puumala virus (PUUV) infections in Germany
Source: PeerJ. 2018 Feb 1;6:e4255. doi: 10.7717/peerj.4255 (PMC5797684; doi:10.7717/peerj.4255)

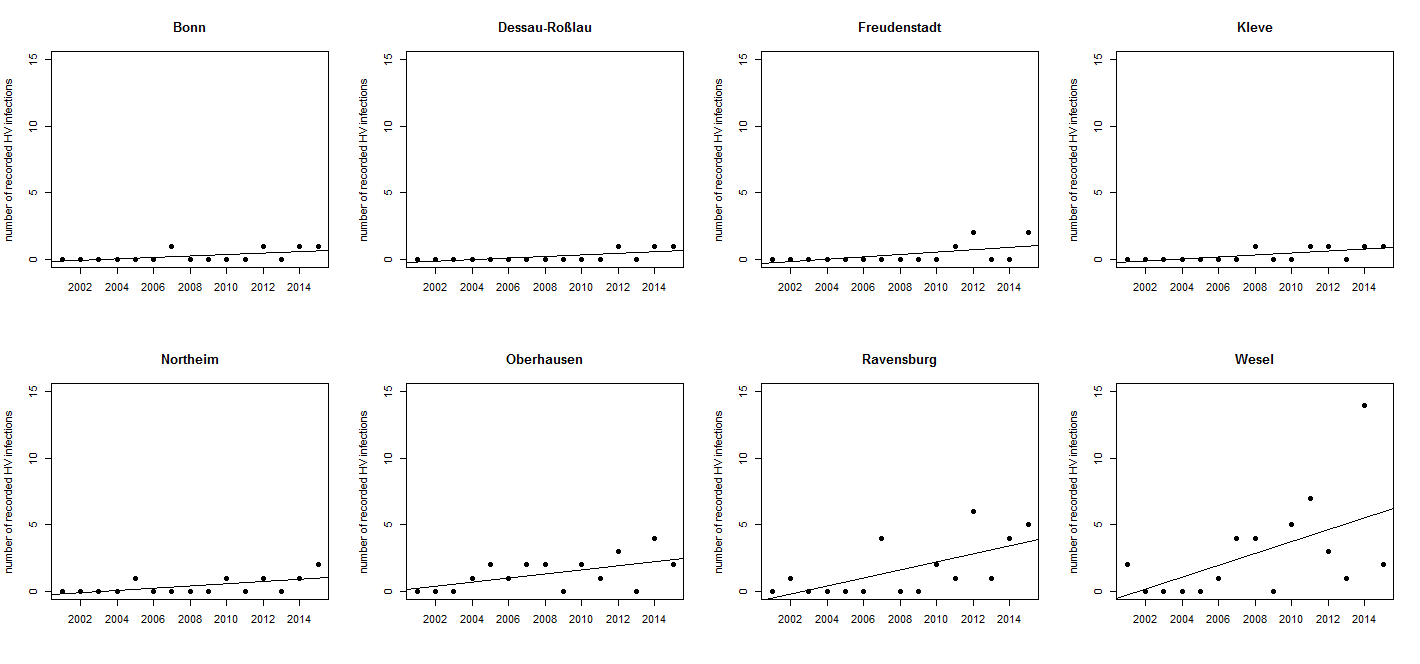

Supplement: Figure S1 [file peerj-06-4255-s001.png]

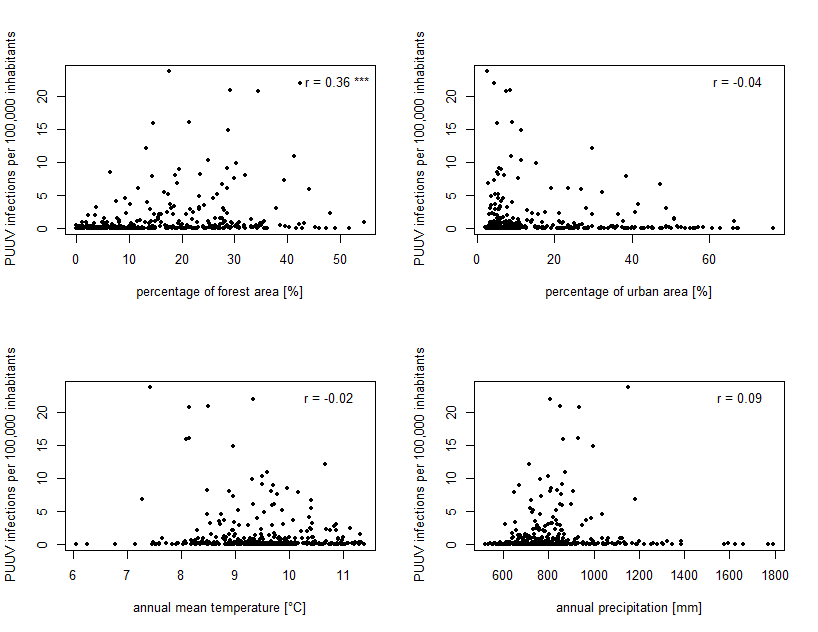

Supplement: Figure S2 — Spearman correlation coefficients (r) between the number of recorded PUUV infections per 100,000 inhabitants and the percentage of forest area as well as the percentage of urban area (derived from the CORINE landcover data) and the annual mean temperature (mean over 2001–2015) as well as annual precipitation (mean over 2001–2015) *** very highly significant (p < 0.001). [file peerj-06-4255-s002.png]
